# Supplementary figures and images for: Effects of Dietary Microcapsule Sustained-Release Sodium Butyrate on the Growth Performance, Immunity, and Gut Microbiota of Yellow Broilers
Source: Animals (Basel). 2023 Nov 21;13(23):3598. doi: 10.3390/ani13233598 (PMC10705809; doi:10.3390/ani13233598)

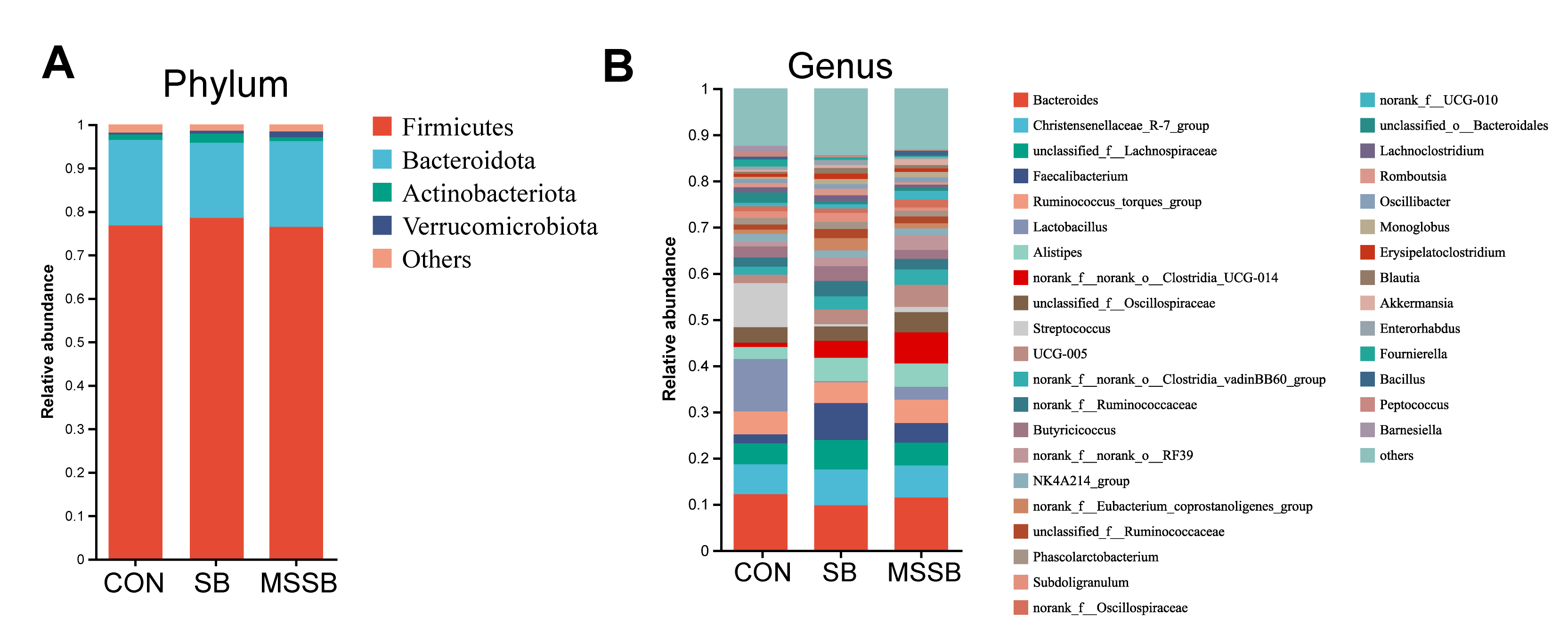

Supplement: Supplementary file 1 [file animals-13-03598-s001.zip › Figure S1.tif]
